# Activity of Bacteriophage and Complex Tannins against Biofilm-Forming Shiga Toxin-Producing *Escherichia coli* from Canada and South Africa

**DOI:** 10.3390/antibiotics9050257

**Published:** 2020-05-15

**Authors:** Emmanuel W. Bumunang, Collins N. Ateba, Kim Stanford, Yan D. Niu, Y. Wang, Tim A. McAllister

**Affiliations:** 1 Department of Microbiology, Faculty of Natural and Agricultural Sciences, Mafikeng Campus, North-West University, Private Bag X2046, Mmabatho 2735, South Africa; bumunang@gmail.com (E.W.B.); Collins.Ateba@nwu.ac.za (C.N.A.); 2 Agriculture and Agri-Food Canada, Lethbridge Research and Development Centre, Lethbridge, AB T1J 4B1, Canada; yuxi.wang@canada.ca; 3 Alberta Agriculture and Forestry, Lethbridge, AB T1J 4V6, Canada; kim.stanford@gov.ab.ca; 4 Department of Ecosystem and Public Health, Faculty of Veterinary Medicine, University of Calgary, Calgary, AB T2N 1N4, Canada

**Keywords:** biofilms, bacteriophage, condensed tannin, phlorotannins, Shiga toxin-producing *Escherichia coli*, stainless-steel coupon

## Abstract

Bacteriophages, natural killers of bacteria, and plant secondary metabolites, such as condensed tannins, are potential agents for the control of foodborne pathogens. The first objective of this study evaluated the efficacy of a bacteriophage SA21RB in reducing pre-formed biofilms on stainless-steel produced by two Shiga toxin-producing *Escherichia coli* (STEC) strains, one from South Africa and the other from Canada. The second objective examined the anti-bacterial and anti-biofilm activity of condensed tannin (CT) from purple prairie clover and phlorotannins (PT) from brown seaweed against these strains. For 24-h-old biofilms, (O113:H21; 6.2 log_10_ colony-forming units per square centimeter (CFU/cm^2^) and O154:H10; 5.4 log_10_ CFU/cm^2^), 3 h of exposure to phage (10^13^ plaque-forming units per milliliter (PFU/mL)) reduced (*p* ≤ 0.05) the number of viable cells attached to stainless-steel coupons by 2.5 and 2.1 log_10_ CFU/cm^2^ for O113:H21 and O154:H10, respectively. However, as biofilms matured, the ability of phage to control biofilm formation declined. In biofilms formed for 72 h (O113:H21; 5.4 log_10_ CFU/cm^2^ and O154:H10; 7 log_10_ CFU/cm^2^), reductions after the same duration of phage treatment were only 0.9 and 1.3 log_10_ CFU/cm^2^ for O113:H21 and O154:H10, respectively. Initial screening of CT and PT for anti-bacterial activity by a microplate assay indicated that both STEC strains were less sensitive (*p* ≤ 0.05) to CT than PT over a concentration range of 25–400 µg/mL. Based on the lower activity of CT (25–400 µg/mL), they were not further examined. Accordingly, PT (50 µg/mL) inhibited (*p* ≤ 0.05) biofilm formation for up to 24 h of incubation at 22 °C, but this inhibition progressively declined over 72 h for both O154:H10 and O113:H21. Scanning electron microscopy revealed that both SA21RB and PT eliminated 24 h biofilms, but that both strains were able to adhere and form biofilms on stainless-steel coupons at longer incubation times. These findings revealed that phage SA21RB is more effective at disrupting 24 than 72 h biofilms and that PT were able to inhibit biofilm formation of both *E. coli* O154:H10 and O113:H21 for up to 24 h.

## 1. Introduction

Food safety is a top priority for the food-processing industry given that foodborne infections are a great burden to public health and also cause huge economic losses [1]. Food products contaminated with Shiga toxin-producing *Escherichia coli* (STEC) have caused serious outbreaks in humans for decades [2,3,4]. For example, STEC serotype O113:H21, which has been associated with human illness [5,6] and O154:H10, which has the ability to colonize and form biofilms on food contact surfaces under different environmental conditions, also poses a health risk [7,8]. The persistence of STEC biofilms on food contact surfaces is a contributing factor to the contamination of food products [9,10].

The potential of biofilms to resist antimicrobial treatment is due to a protective outer layer, the exopolysaccharide matrix and the multiple bacterial layers of biofilms which protect embedded cells against disinfectants and other antimicrobial agents [11,12]. The ability to chelate cationic antimicrobials as a result of the presence of extracellular DNA within the biofilm matrix [13] and to concentrate enzymes such as beta-lactamases, increases the resistance of biofilms to antimicrobials [14]. Additionally, cells within the biofilm enter a state of metabolic dormancy which further reduces the effectiveness of antimicrobials [15]. Furthermore, close proximity of cells within the biofilm can promote the horizontal transfer of genes that confer resistance to antimicrobials [11].

Controlling biofilms is a vital aspect of ensuring food safety within food-processing environments [16] and one of the prime reasons as to why food contact surfaces are routinely sanitized [17]. However, there are safety concerns surrounding the use of antimicrobials in controlling biofilms as it can promote resistance [18] as well as introduce antimicrobial residues into food [19]. Therefore, there is a need for alternative agents such as bacteriophages, which are natural predators of bacteria [20]. 

Bacteriophages (phages) are ubiquitous in nature, host-specific, non-toxic and self-replicating, making them potential alternatives to traditional antimicrobials [21]. Phages could disrupt biofilms through their ability to infect and lyse bacteria. In addition, phages that possess genes coding for exopolysaccharide depolymerases are considered superior anti-biofilm agents as these enzymes can degrade the exopolysaccharide matrix that protects bacteria within biofilms [22,23]. Phages have been applied against bacterial biofilms formed on a variety of different surfaces [24,25,26] and have shown promise as an effective control measure [27,28].

Additionally, the search for novel antimicrobial agents that prevent the adhesion of bacterial cells to surfaces could be instrumental in inhibiting biofilm formation [29,30]. Tannins are polyphenolic compounds with demonstrated anti-bacterial [31,32,33] and anti-biofilm activity [34,35,36]. Although the affinity of tannins for proteins has been well documented [37,38], the precise mechanisms whereby they inhibit STEC and disrupt biofilms is poorly understood. Possible mechanisms include the binding of tannins to bacterial outer membrane proteins and inhibition of cell wall and nucleic acid synthesis [29,33].

This study assessed alternative approaches to controlling biofilms formed by O154:H10 and O113:H21 STEC on stainless-steel coupons. Preliminary investigations found that tannins formed complexes with phage, eliminating their biological activity. As a result, the activity of phage and tannins against biofilms was assessed separately. The first objective was to evaluate the efficacy of a newly isolated depolymerase-secreting phage, SA21RB [39], in removing pre-formed biofilms of these two strains. The second objective was to investigate the activity of condensed tannins from purple prairie clover (*Dalea purpurea*) and phlorotannins from brown seaweed (*Ascophyllum nodosum*) against the formation of biofilms on the surface of stainless-steel coupons.

## 2. Results

### 2.1. Experiment 1

#### 2.1.1. One-Step Growth Curve, and Susceptibility of O154:H10 and O113:H21 to Phage SA21RB

**Figure 1:**
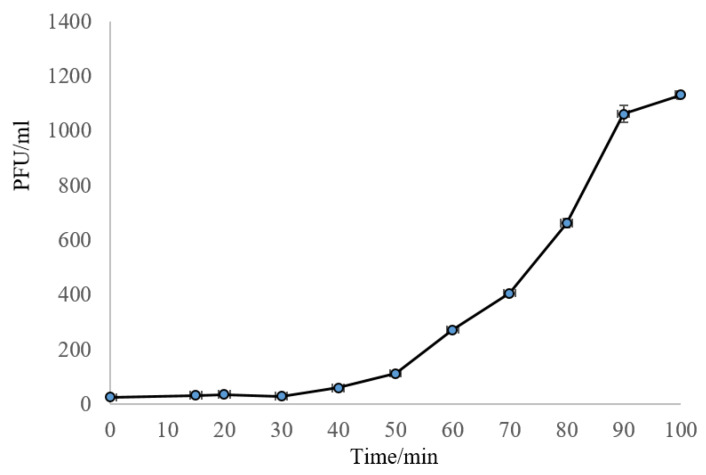
One-step growth curve of phage SA21RB.

The one-step growth curve indicated that phage SA21RB had a latent period of 40 min and a burst size of 43 phages per infected cell (Figure 1). Based on visual observation of a microplate phage virulence assay, both strains (O154:H10 and O113:H21) were susceptible to SA21RB (Table 1).

#### 2.1.2. Treatment of Biofilms with Bacteriophage SA21RB Plate Count and Scanning Electron Microscopy (SEM)

**Figure 2:**
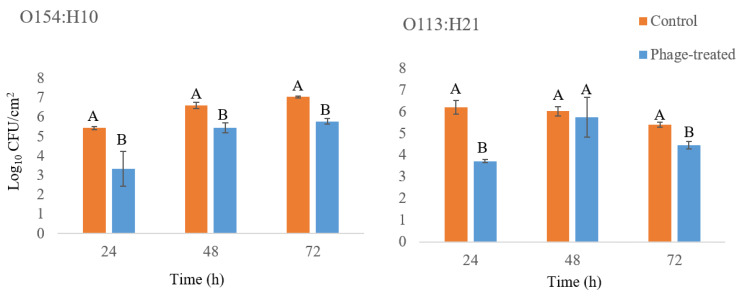
The number of surviving *E. coli* O154:H10 and O113:H21 cells (log_10_ CFU/cm^2^) associated with biofilms treated or untreated with phage SA21RB (10^13^ PFU/mL). Different uppercase letters denote differences at *p* < 0.05 among phage-treated and control coupons. Bars = standard error (*n* = 3).

Removal of biofilms following phage (10^13^ plaque-forming units per milliliter (PFU/mL)) treatment was assessed by enumerating viable cells on stainless-steel after 3 h of treatment at 22 °C, as compared to untreated controls. Results indicated that phage treatment of O154:H10 and O113:H21 reduced (*p* ≤ 0.05) biofilm formation after 24 h, as compared to untreated biofilms (5.4 and 6.2 log_10_ colony-forming units per square centimeter (CFU/cm^2^); Figure 2). Interestingly, the effectiveness whereby phage reduced cell viability decreased (*p* ≤ 0.05) for both *E. coli* strains as biofilms aged. For O113:H21 and O154:H10 respectively, the reductions as a result of exposure to phage were 2.5 and 2.1 log_10_ CFU/cm^2^ for 24 h biofilms as compared to 0.9 and 1.3 log_10_ CFU/cm^2^ for biofilms that had formed for 72 h (Figure 2).

**Figure 3:**
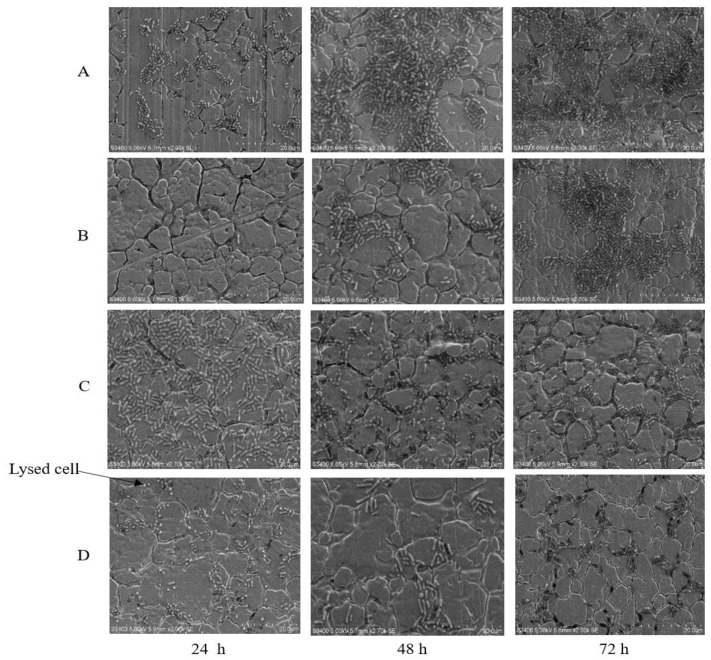
Scanning electron microscopy images for phage-treated (10^13^ PFU/mL) biofilms after 24, 48 and 72 h of incubation. *E. coli* O154:H10 untreated controls (**A**) and phage-treated (**B**), and *E. coli* O113:H21 untreated controls (**C**) and phage-treated (**D**). Bar = 20 µm.

Phage-treated and untreated biofilms were examined by transmission electron microscopy. Results confirmed that phage were more effective against biofilms that formed over 24 h as compared to those that formed over 72 h (Figure 3). Evidence of biofilm cell disruption could be seen in O113:H21, which displayed both lysed and morphologically normal rod-shaped cells (Figure 3).

### 2.2. Experiment 2

#### 2.2.1. Effects of Condensed Tannins (CT) and Phlorotannins (PT) on Growth of O154:H10 and O113:H21 

**Figure 4:**
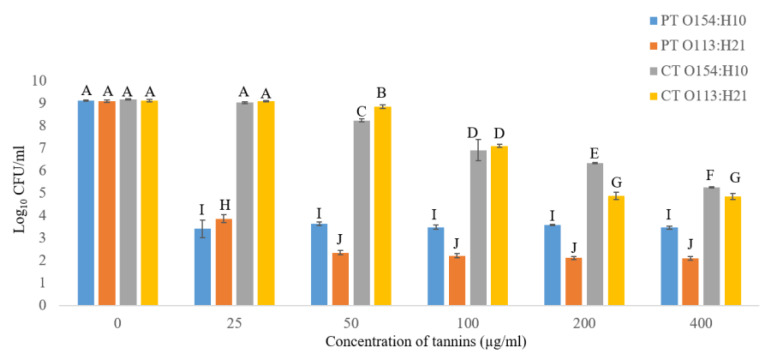
The anti-bacterial effect of condensed tannins (CT) and phlorotannins (PT) at five concentrations (25–400 µg/mL) on *E. coli* O154:H10 and O113:H21. Different uppercase letters denote differences at *p* < 0.05 among condensed tannins and phlorotannins. Bars = standard error (*n* = 3).

Inhibitory effects (*p* ≤ 0.05) of CT and PT on growth of O154:H10 and O113:H21 after 24 h of incubation at 22 °C were observed (Figure 4). PT had a dose-independent action, as similar numbers of viable cells were observed regardless of increasing tannin concentrations (25–400 µg/mL). In contrast, CT showed a dose-dependent effect in which the higher the tannin concentration, the greater the inhibition (Figure 4). Overall, O154:H10 and O113:H21 were more sensitive (*p* ≤ 0.05) to PT than CT, and O113:H21 was more sensitive (*p* ≤ 0.05) to PT than was O154:H10 (Figure 4). CT at the highest concentration (400 µg/mL) had a minimal inhibitory effect (*p* ≤ 0.05), as compared to PT at 25–400 µg/mL (Figure 4). Based on this observation, the ability of PT (50 µg/mL) to inhibit biofilms was investigated and CT were not examined further.

#### 2.2.2. Anti-Biofilm Activity of Phlorotannins

**Figure 5:**
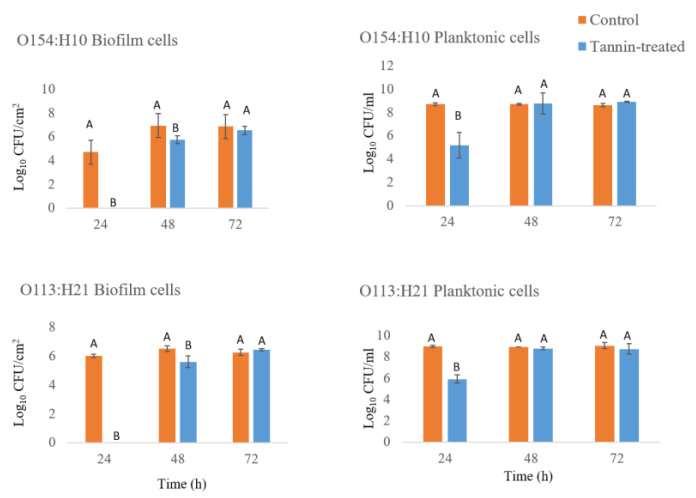
Effect of phlorotannins (50 µg/mL) on *E. coli* O154:H10 and *E. coli* O113:H21 biofilm formation and planktonic cell growth after 24, 48 and 72 h of exposure. Different uppercase letters denote differences at *p* < 0.05 among phlorotannin-exposed and controls. Bars = standard error (*n* = 3).

Biofilm formation was inhibited (*p* ≤ 0.05) by 50 µg/mL of PT for up to 24 h of incubation for O154:H10 and O113:H21 in relation to untreated controls (Figure 5). Similarly, compared to controls (8.7 and 8.9 log_10_ CFU/mL), growth of planktonic cells of O154:H10 and O113:H21 was inhibited (*p* ≤ 0.05) by PT (5.2 and 5.9 log_10_ CFU/mL) after 24 h of incubation (Figure 5). Conversely, both *E. coli* strains overcame the inhibitory effects of PT after 72 h of incubation, as biofilm cell concentrations (6.5 and 6.4 log CFU/cm^2^) were, by that time, similar to those of untreated samples (6.8 and 6.2 log CFU/cm^2^; Figure 5). Likewise, after 48 and 72 h of incubation, growth of planktonic cells from PT-bacteria mixture was similar to untreated controls for both O154:H10 and O113:H21 (Figure 5).

#### 2.2.3. SEM Analysis

**Figure 6:**
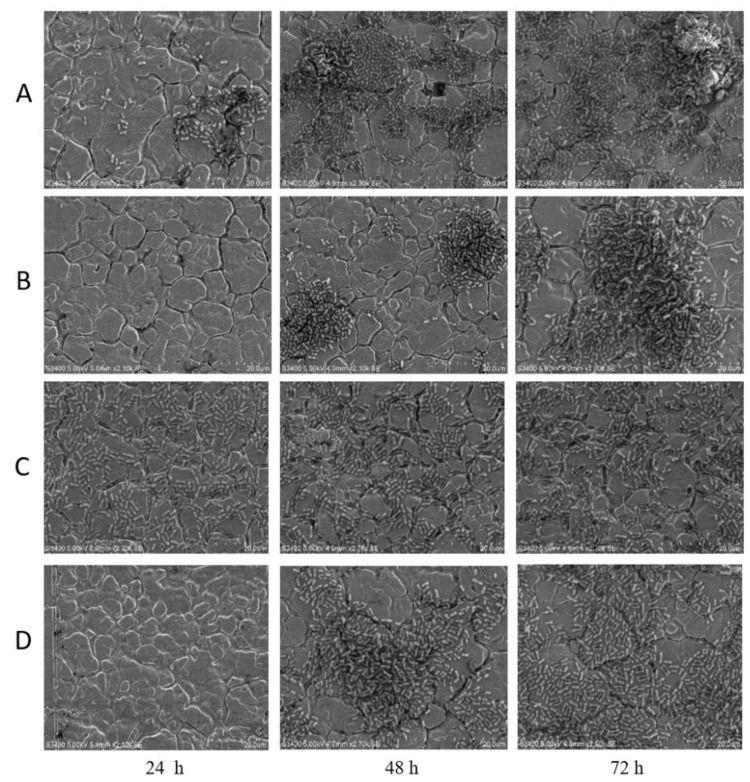
Scanning electron microscopy images for phlorotannins-exposed (50 µg/mL) coupons after 24, 48 and 72 h of incubation. *E. coli* O154:H10 untreated controls (**A**) and phlorotannin-exposed (**B**), and *E. coli* O113:H21 untreated controls (**C**) and phlorotannin-exposed (**D**). Bar = 20 µm.

Compared to controls, no biofilms were found on coupons in PT-bacteria mixture after 24 h of incubation at 22 °C (Figure 6). Nonetheless, O154:H10 and O113:H21 cells were able to grow, adhere and form biofilms on stainless-steel at 48 and 72 h in the presence of PT (Figure 6). No difference in cell morphology was observed with biofilms in PT-bacteria mixture as compared to control biofilms (Figure 6).

### 2.3. Experiment 3

#### Sensitivity of Phage SA21RB to Phlorotannins

The incubation of phage SA21RB and PT for 3 h at 22 °C decreased the viability of phage titer by 9 log_10_ (Table 2; Figure S1 in the Supplementary Material).

## 3. Discussion

### 3.1. Biofilm Removal Using Phage SA21BR

This study evaluated the effectiveness of phage SA21RB and phlorotannins against biofilms formed on stainless-steel by STEC strains O154:H10 and O113:H21 STEC from South Africa and Canada, respectively. Bacteria within biofilms are more resistant to disinfectants and other antimicrobials than those that are in a planktonic form [40,41]. Therefore, removal or prevention of biofilm formation would help curb contamination and dissemination of biofilm-related infectious agents within the food industry [42]. The first part of this study focused on phage–biofilm interactions. Phage SA21RB was selected for this study because of its lytic potential, high burst size and de-polymerase activity [39].

The findings indicated that the susceptibility of O154:H10 and O113:H21 *E. coli* planktonic cells to phage SA21RB is isolate-dependent. Similarly, variable susceptibility of different bacterial strains to the same phage has been reported [43,44] and is attributed to differences in receptors on the surface of the bacterial cell [45]. Phage treatment demonstrated that biofilms of O154:H10 and O113:H21 were susceptible to phage SA21RB and corroborates previous studies which demonstrate that biofilms of different bacterial strains on various food production surfaces are susceptible to phage. For example, a lytic bacteriophage (BPECO 19) was shown to reduce *E. coli* O157:H7 biofilms by 3 log_10_ on stainless-steel [46]. Biofilms of *Listeria monocytogenes* on stainless-steel were reduced by 3.5 to 5.4 log/cm^2^ using bacteriophage P100 [47]. Also, phage φIBB-PF7A was able to reduce *Pseudomonas fluorescens* biofilms on stainless-steel coupons by 3 to 5 log_10_ after 4 h of phage exposure [48]. However, as in our study, biofilms were not completely removed from stainless-steel coupons as a result of exposure to phage. Factors such as temperature [49], cell density [50], diffusion barrier [51] and bacterial growth stage [52] can affect the extent that biofilm cells are lysed by phages. In this study, 3 h of exposure to phage SA21RB resulted in partial removal of biofilms at 22 °C. This treatment duration was selected given that phage SA21RB showed a latent time of 40 min, and also to reduce the likelihood of phage-resistant bacterial strains emerging as a result of longer exposure periods [48]. Effectiveness of the phage against biofilms formed by both strains (O154:H10 and O113:H21) decreased with biofilm aging. A greater log reduction in cells associated with 24 h as compared to 72 h biofilms could reflect the greater metabolic activity of cells in younger biofilms, increasing their susceptibility to phage. In contrast, cells in more mature biofilms are less metabolically active and less susceptible to phage infection given their embedded location within the biofilm matrix [53]. Another possible explanation, reviewed by Harper, Parracho, Walker, Sharp, Hughes, Werthén, Lehman and Morales [54], suggested that phage can infect less metabolically active cells but cannot replicate, as the expression of the lytic gene is suppressed in cells that are in stasis as compared to those that are actively growing. Based on our data, a decrease in the rate of biofilm removal by phage SA21RB can be attributed to the metabolic state of the biofilm cells and not to the number of cells on the coupon, as numbers were similar at 24 and 72 h. Consequently, phage SA21RB was most likely to be effective if it was used to control newly formed biofilms.

Some phage possess enzymes that can degrade the exopolysaccharide (EPS) components of the biofilm matrix [55]. Phage SA21RB showed de-polymerase activity against O154:H10 planktonic cells [39], suggesting that the failure of the phage to completely remove the biofilm is unlikely due to its inability to penetrate the EPS barrier. Therefore, this observation supports the suggestion that complete eradication of biofilms using bacteriophage is unlikely [27]. However, the promising effects of phage in reducing biofilms on stainless-steel suggest that they could be used at some point after the application of disinfectants so as to target recently formed biofilms.

### 3.2. Biofilm Prevention Using Phlorotannins

Incomplete eradication of biofilms of these strains using phage SA21RB motivated us to explore tannins for their anti-biofilm activity. The anti-bacterial activity of condensed tannins from purple prairie clover and phlorotannins from brown seaweed, against planktonic O157 and non-O157 *E. coli*, has been examined previously [31]. Wang et al [31] indicated that PT had a greater effect against non-O157 *E. coli* than terrestrial CT. Similarly, this work found that PT have greater activity against planktonic cells of O154:H10 and O113:H21 than CT. Greater anti-bacterial activity of PT has been attributed to their high degree of polymerization [56,57]. In addition, PT readily forms complexes with proteins through free hydroxyl group [58], and have more of these reactive sites than CT [31]. Variation in the nature of outer membrane proteins may also account for the differences in the sensitivity of O113:H21 and O154:H10 to PT, an observation that corroborates the findings of others [31,56]. The anti-bacterial activity of tannins is attributed to several mechanisms, including its ability to complex with the outer membrane of bacteria and the sequestration of minerals required for bacterial growth [58]. Therefore, it was assumed that the ability of PT to inhibit planktonic growth of O154:H10 and O113:H21 could stop biofilm formation.

Our results indicate that PT could stop the initiation of biofilms by planktonic O113:H21 and O154:H10 over a period of up to 24 h. Similar results were reported by Trentin et al [35], who found that CT from *Anadenanthera colubrine*, *Commiphora leptophloeos* and hydrolyzable tannins from *Myracrodruon urundeuva* inhibited biofilm formation by *Pseudomonas aeruginosa* on polystyrene over 24 h. Also, the report of da Silva et al [59] indicates that polyphenolic compounds in medicinal plants from Brazil have an anti-biofilm effect against *Staphylococcus epidermidis* in polystyrene 96-well microtiter plates over 24 h.

The mechanisms responsible for the effect of plant polyphenols on biofilm formation are not fully understood. Some possibilities include inhibition of cell growth and exopolysaccharide synthesis, anti-adhesivity and attachment activities and inhibition of quorum sensing [29,60]. The former supports the explanation as to why no biofilm cells were obtained after 24 h in this study and can be attributed to minimal planktonic bacterial growth in the presence of PT. Additionally, an increase in the planktonic cell number with incubation > 24 h corroborates the biofilm results after 48 h in the presence of PT, showing growth above the minimum range (≥5 log CFU/cm^2^) indicative of biofilm formation [61]. Therefore, another explanation could be that PT inhibit, but do not completely stop, the growth of STEC. As STEC continue to grow, they eventually produce enough cells to inactivate the PT, enabling biofilms to form at later incubation times. Contrary to this hypothesis, Trentin et al [35] found that tannins inhibited biofilm formation without inhibiting bacterial growth. For example, da Silva et al [59] observed that plant extracts of *Bauhinia acuruana*, *Commiphora leptophloeos*, *Bauhinia acuruana* and *Pityrocarpa moniliformis* prevented biofilm formation by *Staphylococcus epidermidis* on glass and polystyrene without inhibiting planktonic cell growth. These reports propose that the anti-adhesive properties of the plant extracts are responsible for their anti-biofilm activity. A previous study has demonstrated that the rate of inactivation of tannin is higher at 20 or 37 °C than at 0 °C [62]. Also, PT contain many hydroxyl groups and are highly oxidized when exposed to air [63,64]. The loss of activity in PT in this study could be a result of oxidation of these groups through exposure to air at 22 °C.

Based on SEM results, no biofilm cells were observed after 24 h of exposure to PT, an observation that was confirmed by plate counts of PT-exposed cells. Nevertheless, attached bacterial cells were enumerated after 48 and 72 h and exhibited a normal rod-shaped morphology in the presence of PT. Another possible explanation for bacterial attachment and subsequent biofilm formation could be that PT did not inhibit or interact with extracellular structures involved in biofilm formation, such as the fimbriae and curli [65]. Also, the normal rod-shaped morphology suggests there was no direct interaction of PT with the bacterial cell membrane. This observation is contrary to a study which found that some *P. aeruginosa* biofilm cells exhibited altered morphology as a result of exposure to CT [35]. The differences in these observations corroborates the views that polyphenols from different plant sources [12] differ in their biological activity [66] at varying concentrations [67].

### 3.3. Inactivation of Phage SA21RB by Phlorotannins

Tannins can inactivate viruses by their ability to bind and interact with viral proteins [68]. The reduction in phage SA21RB titer (by 9 log_10_) in the presence of PT corroborates that of Kulikov et al [69], where viable *E. coli* phage in a phage-tannins (from black tea leaves) mixture were reduced from 10^10^ PFU/mL to 10^1^ PFU/mL after exposure for 10 min. Antiviral activity of tannins has been reported [68,70]. Although there is little information on the mechanisms of action, it is suggested that tannins can irreversibly bind to the structural proteins (capsid and adhesins) responsible for the adsorption of phage to host receptors. Therefore, it can be speculated that the reduction in SA21RB titer might have resulted from PT interacting with phage SA21RB in a manner that impedes its ability to attach to bacterial host receptors. In fact, a mixture of tea leave extracts and ferrous sulfate have been used to inactivate *E. coli* phages [71] and *Salmonella typhimurium* phage [72]. Therefore, PT would have likely impaired the ability of a phage-PT mixture to control biofilms on stainless-steel.

## 4. Materials and Methods

### 4.1. Origin of Strains and Preparation

*E. coli* O154:H10 is a multidrug-resistant (streptomycin, tetracycline, ampicillin, chloramphenicol, trimethoprim-sulfamethoxazole, nalidixic acid and norfloxacin) strain isolated from South African cattle feces [7] and EC20020170. O113:H21 is a Canadian STEC, of human origin, provided by Dr. Roger Johnson of the Public Health Agency of Canada (Guelph, ON). These two strains were selected based on their strong biofilm-forming abilities on polystyrene [7,73] and stainless-steel [8,74]. The strains were streaked from glycerol stocks stored at −80 °C onto Lysogeny broth (LB) agar (Sigma-Aldrich, Oakville, Ontario, Canada) and incubated aerobically at 37 °C for 18 h.

### 4.2. Experiment 1

#### 4.2.1. Bacteriophage Preparation and Titration

Phage SA21RB was isolated from cattle feces in the North-West province of South Africa [39]. Phage SA21RB was chosen because it can infect and lyse *E. coli* O154:H10 and O113:H21 and exhibits de-polymerase activity. Phage SA21RB was propagated using *E. coli* O154:H10 in minimal salt (M9) broth (Sigma-Aldrich) supplemented with 0.4% (*w*/*v*) glucose, 0.02% (*w*/*v*) MgSO_4_ and 0.001% (*w*/*v*) CaCl_2_, as described in Reference [75]. Briefly, 150 µL of diluted phage stock preparation (10^9^ PFU/mL) and 1.5 mL of mid-log bacterial culture were mixed and incubated for 15 min at 37 °C with gentle shaking at 130 rpm. After incubation, the bacteria/phage mixture was added to 150 mL of M9 medium and incubated overnight at 37 °C with shaking at 170 rpm. The overnight bacteria/phage mixture was centrifuged (Beckman Coulter, Brea, CA, USA) at 4750× *g* for 40 min at 4 °C and filter sterilized using a sterile 500 mL disposable vacuum filtration unit (0.22 µm, Nalgene^®^ Rapid-Flow^Tm^ Filter, Thermo Scientific, West Sacramento, CA, USA). Bacteriophage titer was evaluated by the soft agar overlay plaque assay [76]. The phage SA21RB solution used in the biofilm assay contained 10^13^ PFU/mL.

#### 4.2.2. One-Step Growth Curve

A one-step growth curve was generated as described by Ellis and Delbrück [77]. Briefly, 100 µL of phage (10^6^ PFU/mL) was added to 900 µL overnight host (O154:H10) culture and incubated for 10 min at 37 °C. After incubation, the phage-bacterial mixture was diluted by adding 100 µL to 9.9 mL of LB in a 15 mL Falcon tube. Subsequently, 100 µL of diluted phage-bacterial solution was transferred to another tube containing 9.9 mL of LB and incubated in a water bath at 37 °C. The phage-bacterial mixture was sampled (100 µL) every 10 min over a period of 100 min and assessed in a plaque assay. For the plaque assay, 100 µL of the phage-bacterial mixture was mixed with 3 mL of 0.3% (*w*/*v*) molten top agar and overlaid onto LB plates. The first sample was plated immediately (time zero) and after 15 min of incubation. From the 20th min of incubation, samples were plated every 10 min. Plate counts were performed in triplicate and plotted to obtain the one-step growth curve.

#### 4.2.3. Microplate Phage Virulence Assay

The susceptibility of O154:H10 and O113:H21 *E. coli* to phage SA21RB was determined as described by Niu et al [44]. Briefly, 20 µL of phage SA21RB was 10-fold serially diluted in 180 µL M9 broth in a 96-well microplate (Nunc, Edmonton, AB, Canada). Wells were then inoculated with 20 µL of overnight cultures of O154:H10 and O113:H21 diluted 1:10 in M9. Microplates were incubated at 22 °C for 18 h. Duplicate wells were inoculated for each bacterial strain. A negative control (wells containing M9 broth only) and a positive control (wells containing M9 broth, bacteria and no phage) were included. After incubation, phage susceptibility to bacteria was determined by visual observation of wells for turbidity as a result of bacterial growth.

#### 4.2.4. Biofilm Removal Using Phage SA21RB 

Biofilms were formed as described by Ma et al [74]. For the phage treatment study, biofilms were formed on stainless-steel coupons (type 304 with number 2b finish 2.54 × 3.81 × 0.081, Biosurface, Bozeman, MT, USA) for 24, 48 or 72 h at 22 °C. Subsequently, the coupons were placed into a disposable sterile petri dish (60 × 15 mm) containing 5 mL of 10^13^ PFU/mL phage suspension or M9 medium (without glucose) as a control and incubated for 3 h at 22 °C. After 24, 48 or 72 h, coupons with formed biofilms were placed in 25 mL of sterile distilled water in a 50 mL Falcon tube and rinsed three times in three consecutive tubes. Phage-treated and control coupons were placed in 25 mL of sterile 10 mM phosphate buffered saline (PBS, pH 7.4), and sonicated at 20 kHz for 10 min, followed by vortexing for 1 min. An aliquot (2 mL) of the suspension was placed in an Eppendorf tube and centrifuged for 3 min at 13,000× *g* to separate unabsorbed phage from bacteria. The supernatant was discarded, and bacteria were resuspended in 1 mL of sterile PBS. Bacterial suspension was 10-fold serially diluted, plated on LB agar, and incubated at 37 °C for 18 h. To verify the action of phage SA21RB on preformed biofilms, plate counts of phage-treated bacteria strains were compared with those of a positive control. The results were expressed as the average of the data from two independent assays.

### 4.3. Experiment 2

#### 4.3.1. Isolation of Condensed Tannins and Phlorotannins

Condensed tannins and phlorotannins were extracted from purple prairie clover (*Dalea purpurea*) grown at Lethbridge, AB, Canada, and brown seaweed (*Ascophyllum nodosum*) was harvested from the Atlantic coast of Nova Scotia, Canada (Acadian Seaplants Limited, Dartmouth, NS, USA). Samples were extracted with acetone, water and ascorbic acid (7:3:1), as described by Wang et al [31]. Specifically, 500 g of ground samples were mixed in extraction solvent (70% aqueous acetone *v*/*v*) and stirred for 1 h at room temperature (22 °C). Samples were filtered through a monofilament polyester 335 µm filter and the filtrate was mixed with an equal volume of diethyl ether to achieve phase separation. The supernatant was aspirated using a Speed Vac water jet (Savant Instruments, Inc. Farmingdale, New York, NY, USA). The aqueous phase was centrifuged (20 min; 5000× *g*; 4 °C) and filtrates were rotary evaporated under vacuum at 40 °C to remove acetone and ether. The remaining aqueous residue (crude tannin) was freeze-dried. The freeze-dried powder of condensed tannins (CT) and phlorotannins (PT) were further processed to isolate tannins, as described by Wang et al [31]. Briefly, dried crude tannin extracts were dissolved in 80% (*v*/*v*) ethanol. The mixture was stirred for 1 h at room temperature (22 °C) and filtered through an EMD Millipore Glass Vacuum Filter Holder. Filtrate was added to a Sephadex^TM^ LH-20 (Sigma-Aldrich) slurry equilibrated with 80% (*v*/*v*) ethanol. The tannin-Sephadex mixture was left for 45 min at room temperature to enable tannins to bind to the Sephadex beads. The tannin-Sephadex mixture was filtered using an EMD Millipore Glass Vacuum Filter Holder to remove non-tannin materials. Subsequent washes were performed on tannin-Sephadex with 4750 mL of 80% (*v*/*v*) ethanol until the elute was clear. Thereafter, tannins were eluted from the beads using 2000 mL of 50% acetone. The aqueous acetone-tannin elution was rotary evaporated under vacuum at 40 °C to remove acetone. Purified extracts were freeze-dried and stored at −20 °C in an amber bottle until used.

#### 4.3.2. Effects of Condensed Tannins (CT) and Phlorotannins (PT) on Growth of O154:H10 and O113:H21 *E. coli*

The two tannin extracts were dissolved separately in sterile M9 medium supplemented with 0.4% (*w*/*v*) glucose, 0.02% (*w*/*v*) MgSO_4_ and 0.001% (*w*/*v*) CaCl_2_ to obtain a concentration of 400 µg/mL. Two-fold dilutions of both the CT and PT were obtained in accordance with Wang et al [31], generating tannin concentrations of 400, 200, 100, 50 and 25 µg/mL that were used to assess the minimum inhibitory concentration (MIC) of the extracts. Overnight bacterial culture of O154:H10 and O113:H21 *E. coli* were diluted (1:10) with M9 medium to achieve a final concentration of 7 log CFU/mL. Bacterial inoculum (50 µL) was added to each well within a 96-well microplate (Nunc, Edmonton, AB, Canada) containing 150 µL of the different concentrations of either CT or PT. Triplicate wells were inoculated for each bacterial strain. Negative (wells containing tannin, M9 and no bacteria) and positive controls (wells with M9, bacteria and no tannin) were included. Wells with tannin and M9 medium only were used as blank controls. Plates were incubated aerobically without shaking for 24 h at 22 °C and growth was determined by measuring the optical density at 600 nm using an enzyme-linked immunosorbent assay (ELISA) plate reader (Synergy^TM^ HT BioTek, Highland Park, Winooski, VT, USA). To determine the minimum bactericidal concentration (MBC), 100 µL of varying concentrations (400, 200, 100, 50 and 25 µg/mL) were used in the MIC assay and control wells without tannins were 10-fold serially diluted and plated on LB agar and incubated at 37 °C for 18 h to enumerate colonies.

#### 4.3.3. Anti-Biofilm Assay of Phlorotannins on Stainless Coupons

Biofilm formation potential of the isolates was assessed as described by Ma et al [74]. Compared to PT, CT exhibited a weak inhibitory effect at concentration 25–400 µg/mL on planktonic cells of O154:H10 and O113:H21. Consequently, the anti-biofilm activity of PT at the lowest concentration (50 µg/mL) exhibiting bacteriostatic activity against O154:H10 and O113:H21 was further evaluated. The same stainless-steel coupons as described above were used for anti-biofilm analyses. Specifically, PT was diluted in M9 medium to achieve a final concentration of 50 µg/mL. Thereafter, overnight STEC cultures were diluted in PT-M9 medium to achieve a final concentration of 7 log CFU/mL, coupon immersed into 50 mL Falcon tubes and incubated for biofilm formation, as described by Ma et al. [41]. In the biofilm formation assay, M9 medium without PT was used as a positive control. Data were acquired from two independent experiments conducted in triplicates.

#### 4.3.4. Enumeration of the Planktonic and Adhered Bacterial Cells after Anti-Biofilm Assay

Planktonic cells after each period were enumerated according to Bumunang et al. [8]. Briefly, 1 mL of the cell suspension was pipetted from the Falcon tube (with and without tannin treatment), ten-fold serially diluted with 10 mM PBS (pH 7.4), plated on LB agar and incubated aerobically at 37 °C for 18 h. Subsequently, the colony-forming units (CFU/mL) were determined for each plate from two independent experiments. Biofilm cells on coupons (with and without tannin treatment) were removed according to Bumunang et al. [8]. Briefly, coupons were rinsed three times with sterile water, immersed in 25 mL of sterile PBS and sonicated at 20 kHz for 10 min, followed by vortexing for 1 min. The same procedure as above was used to enumerate biofilm cells. 

### 4.4. Scanning Electron Microscopy (SEM) Analysis

Phage-treated and control coupons, as well as tannin-treated and control coupons, were prepared as described by Ma et al. [41], and biofilm cells were examined by scanning electron microscopy according to Arnold and Bailey [78]. Briefly, treated (phage or tannin) and untreated coupons were fixed in 2.5% (*v*/*v*) glutaraldehyde overnight following a series of ethanol dehydration at 10%, 30%, 50%, 70%, 90% and 100%, and isobutyl alcohol dilutions (*v*/*v*) (i.e., 10%, 30%, 50%, 70%, 90% and 100%). The coupons were then dried in 100% (*v*/*v*) hexamethyldisilazane for 10 min and observed with a scanning electron microscope (HITACHI S-4800, Tokyo, Japan).

### 4.5. Experiment 3

#### Virucidal Action of Phlorotannins against Phage SA21RB

The virucidal property of phlorotannins against phage SA21RB was tested as described by Kulikov et al. [69], with some modifications. Briefly, PT were prepared according to Wang et al [31], and 1 mL of phage stock (10^13^ PFU/mL) was added to 1 mL of phlorotannins (100 µg/mL) to achieve a final concentration of 50 µg/mL. Equally, 1 mL of phage stock was added to 1 mL of M9 broth (PT-free) to serve as a control. Phage-PT mixture was incubated at 22 °C for 3 h, conditions similar to those used in the phage SA21RB biofilm removal study. After incubation, aliquots of the phage-PT mixtures (100 µL) were used for a plaque assay using the soft agar overlay technique [76] to enumerate viable phages. Controls included a mixture of mid-log phase bacteria (O154:H10; host strain) with phage only, without PT, and a mixture of bacteria and M9 only (no phage and PT). Assays were performed in triplicate.

### 4.6. Statistical Analysis

Experiments one and two were independently performed two times with three replicates for phage-treated and non-treated samples, as well as tannin-treated and control samples, according to Bumunang et al. [8]. Data were reported as the average and standard deviation within each triplicate. Difference in treated and untreated samples were determined using the least squares means and two-way analysis of variance (ANOVA) in the Statistical Analysis System (SAS 8.01, SAS Institute, Cary, NC, USA). Statistical significance was presented at *p* ≤ 0.05 and means were separated using the general linear models’ procedure.

## 5. Conclusions

Populations of biofilm cells on stainless-steel coupon were reduced by phage SA21RB, with this response being more apparent for biofilms that formed over 24 h as compared to 72 h. Phage did not completely remove biofilm cells, although we assumed that the use of a depolymerase-producing phage would increase the extent of biofilm removal. Further studies by either applying phage SA21RB as a cocktail with other phages or in synergy with antimicrobials may be needed to enhance activity against STEC biofilms. Phlorotannins prevented biofilm formation for up to 24 h, although this efficacy decreased with longer incubation times. Treatment of stainless-steel surfaces with PT every 24 h could be one approach to reducing the formation of STEC biofilms on stainless-steel in food-processing environments.

## Figures and Tables

**Table 1:** Lytic effect of phage SA21RB on *E. coli* O154:H10 and *E. coli* O113:H21.

Phage Titer (Plaque-Forming Units per Milliliter (PFU/mL))	O154:H10	O113:H21
1 × 10^13^	C	C
1 × 10^12^	SC	C
1 × 10^11^	T	C
1 × 10^10^	T	SC
1 × 10^9^	T	T

C, clear (complete lysis); SC, slightly clear; T, turbid (no lysis).

**Table 2:** Effect of phlorotannins (50 µg/mL) on the number of viable phage after 3 h of exposure at 22 °C.

Dilution	Phage Titer (PFU/mL)	
	Phage Only	Phage-PT
1	TNTC	1.5 × 10^3^
8	TNTC	0
9	TNTC	0
10	8.7 × 10^12^	0

TNTC, too numerous to count.

## Supplementary Materials

The following are available online at https://www.mdpi.com/2079-6382/9/5/257/s1: Figure S1: Effect of phlorotannin (PT; 50 µg/mL) on phage SA21RB using soft agar overlay technique. Bacteria only (no phage and PT (A)); bacteria, phage and PT (dilution 1 (B)); bacteria, phage and PT (dilution 8 (C)); soft agar only (no bacteria, phage and PT (D)); bacteria and phage only (no PT dilution 1 (E)) and bacteria and phage only (no PT dilution 8 (F)).
