# Supplementary material for: Activity of Bacteriophage and Complex Tannins against Biofilm-Forming Shiga Toxin-Producing Escherichia coli from Canada and South Africa
Source: Antibiotics (Basel). 2020 May 15;9(5):257. doi: 10.3390/antibiotics9050257 (PMC7277190; doi:10.3390/antibiotics9050257)
Supplement: Supplementary file 1 [file antibiotics-09-00257-s001.pdf]

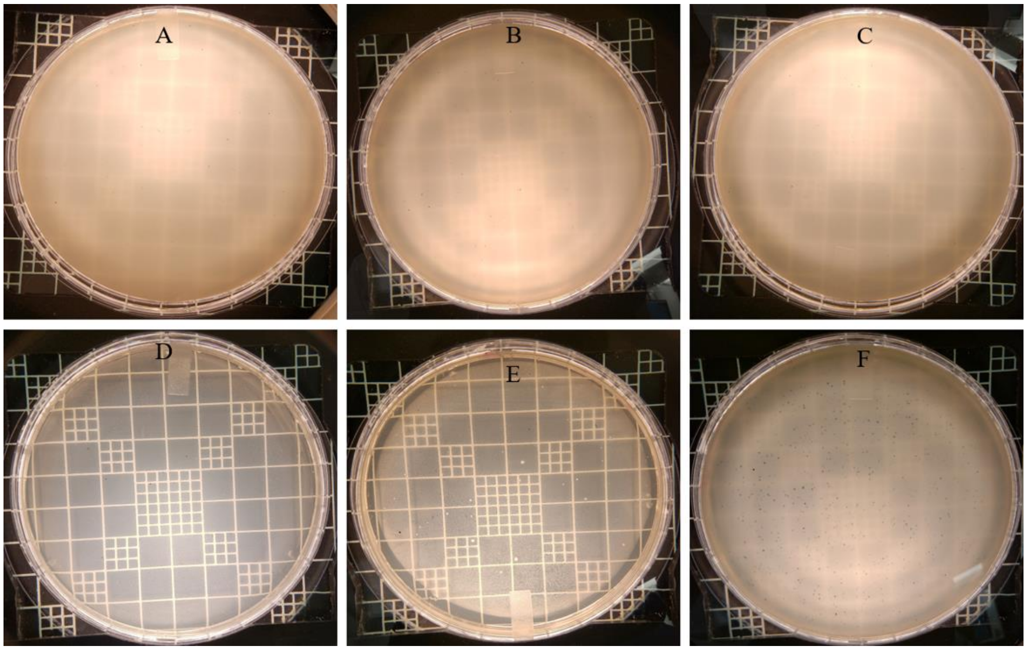

Figure S1. Effect of phlorotannin (PT; 50 μg/mL) on phage SA21RB using the soft agar overlay technique. Bacteria only (no phage and PT (**A**)); bacteria, phage and PT (dilution 1 (**B**)); bacteria, phage and PT (dilution 8 (**C**)); soft agar only (no bacteria, phage and PT (**D**)); bacteria and phage only (no PT dilution 1 (**E**)) and bacteria and phage only (no PT dilution 8 (**F**)).
